# Bayesian Computational Methods for Sampling from the Posterior Distribution of a Bivariate Survival Model, Based on AMH Copula in the Presence of Right-Censored Data

**DOI:** 10.3390/e20090642

**Published:** 2018-08-27

**Authors:** Erlandson Ferreira Saraiva, Adriano Kamimura Suzuki, Luis Aparecido Milan

**Affiliations:** 1 Instituto de Matemática, Universidade Federal de Mato Grosso do Sul, Campo Grande 79070-900, Brazil; 2 Departamento de Matemática Aplicada e Estatística, Universidade de São Paulo, São Carlos 13566-590, Brazil; 3 Departamento de Estatística, Universidade de São Carlos, São Carlos 13565-905, Brazil

**Keywords:** Bayesian inference, Ali–Mikhail–Haq copula, MCMC, Metropolis-Hastings, slice sampling

## Abstract

In this paper, we study the performance of Bayesian computational methods to estimate the parameters of a bivariate survival model based on the Ali–Mikhail–Haq copula with marginal distributions given by Weibull distributions. The estimation procedure was based on Monte Carlo Markov Chain (MCMC) algorithms. We present three version of the Metropolis–Hastings algorithm: Independent Metropolis–Hastings (IMH), Random Walk Metropolis (RWM) and Metropolis–Hastings with a natural-candidate generating density (MH). Since the creation of a good candidate generating density in IMH and RWM may be difficult, we also describe how to update a parameter of interest using the slice sampling (SS) method. A simulation study was carried out to compare the performances of the IMH, RWM and SS. A comparison was made using the sample root mean square error as an indicator of performance. Results obtained from the simulations show that the SS algorithm is an effective alternative to the IMH and RWM methods when simulating values from the posterior distribution, especially for small sample sizes. We also applied these methods to a real data set.

## 1. Introduction

In survival studies, it is common to observe two or more lifetimes for the same client, patient or equipment. For instance, in a bivariate scenario, the lifetimes of a pair of organs can be observed, such as a pair of kidneys, liver, or eyes in patients; or the lifetimes of engines in a twin-engine airplane.

These variables are usually correlated and we are interested in the bivariate model that considers the dependence between them. The copula model is useful for modeling this kind of bivariate data. It has been used in several articles, including the following: [1] describes a comparison between bivariate frailty models, and models based on bivariate exponential and Weibull distributions; [2] proposes a copula model to study the association between survival time of individuals infected with HIV and persistence time of infection; [3] models the association of bivariate failure times by copula functions, and investigates two-stage parametric and semi-parametric procedures; and [4] considers a Gaussian copula model and estimates the copula association parameter using a two-stage estimation procedure.

According to [5,6], a copula is a joint distribution function of random variables for which the marginal probability distribution of each variable is uniformly distributed on the interval [0,1].

There are many parametric copula families in the literature, each one representing a different dependence structure between the random variables. One advantage of a copula model is its simplicity when applied to model bivariate data. This is explored by many authors in survival analysis. Among them are: Romeo et al. [7] and da Cruz et al. [8], who considered the Archimedean copula family; Louzada et al. [9] and Suzuki et al. [10], who considered the Farlie–Gumbel–Morgenstern (FGM) copula; and Romeo et al. [11], who considered the two-parameter Archimedean family of power variance function (PVF) copulas.

In this paper, we apply the Ali–Mikhail–Haq (AMH) copula to model bivariate survival data with random right-censored observations. From a practical point of view, the main reason for using the AMH copula is that it is an Archimedean copula that allows both positive and negative values for the dependence parameter, and whose mathematical formula is simpler than other Archimedean copulas. Another advantage is that assuming the AMH copula, the Kendall rank-order correlation τ between the bivariate lifetimes is a monotonic function of the dependence parameter ϕ. According to [12], the Kendall’s τ can range from (approximately) −0.18 to 0.33, with τ=0 when ϕ=0; and the Spearman’s ρ associated to ϕ can range (approximately) from −0.2711 to 0.4784, indicating that the AMH copula is adequate for modeling bivariate data with a weak correlation.

In order to proceed with the copula model it is necessary to specify the marginal distributions. At this point, several probability distributions could be considered. Generally, the choice for marginal distributions depends on the application. We restrict our analysis to the case where the marginal distributions are Weibull distributions. This is because it is a very flexible distribution for the modeling of various types of lifetime data. In addition, the parametrization of the Weibull distribution—as well as the mathematical expression of the AMH copula—is very attractive from the mathematical point of view, allowing the development of a Bayesian approach to estimate the parameters of interest in a clear and concise way.

As the conditional posterior distributions for parameters of interest does not follow any familiar distribution, the estimation procedure was carried out using versions of the Metropolis–Hastings algorithm, referred to here as Independent Metropolis–Hastings (IMH), Random Walk Metropolis (RWM) and Metropolis–Hastings (MH). MH refers to the Metropolis–Hastings algorithm with a natural candidate generating density whose parameters depend on the hyperparameter values and the observed data. Since the creation of a good candidate generating density in IMH and RWM can be difficult, we also used the slice sampling algorithm [13].

Combining IMH, RWM, MH and SS in different ways, we developed three MCMC algorithms to estimate the model parameters. A simulation study was carried out with the objective of investigating the behavior of each algorithm. The data sets were generated by considering different sample sizes and percentages of right-censored observations. Based on the root mean square error (RMSE), we identified the algorithms with the best performances when estimating the model parameters. We also compared the performances of the three algorithms using the effective sample size and the integrated autocorrelation time [14]. Results obtained from these simulations show that the algorithm that applied the SS algorithm is an effective alternative for standard MCMC methods (IMH and RWM) when simulating values from the posterior distribution of the model parameters, especially when the sample size is small.

We applied the three proposed algorithms to a real data set. This data set is related to diabetic retinopathy, described in The Diabetic Retinopathy Study Research Group [15], and is available in the `survival’ package [16] of the R software [17]. For this case, we compared the performance of the algorithms. Comparison was based on the RMSE relative to the empirical distribution function obtained from Kaplan–Meier estimates.

The remainder of the paper is organized as follows. In Section 2, we introduce the bivariate survival model based on the AMH copula with Weibull marginal distributions. The Bayesian approach and the three MCMC algorithms are described in Section 3. In Section 4, the simulation study is reported. In Section 5 we apply the three algorithms to the real data set. Section 6 summarizes our findings.

## 2. Bivariate Survival Model and Observed Data

Let $\left(T_{1},T_{2}\right)$ be the vector of bivariate lifetimes of an item (or an individual) with marginal density functions $\left(f\left(t_{1}|\theta_{1}\right),f\left(t_{2}|\theta_{2}\right)\right)$ and the survival functions be $\left(S\left(t_{1}|\theta_{1}\right),S\left(t_{2}|\theta_{2}\right)\right)$, where $\theta_{1}$ and $\theta_{2}$ are unknown parameters (scalars or vectors).

Consider that $\left(T_{1},T_{2}\right)$ comes from the copula ${\tilde{C}}_{\phi }$, where ϕ is a parameter showing dependence between $T_{1}$ and $T_{2}$. Then the joint survival function for $\left(T_{1},T_{2}\right)$ is given by

$$S\left(t_{1},t_{2}|\theta ,\phi \right)={\tilde{C}}_{\phi }\left(S_{1}\left(t_{1}|\theta_{1}\right),S_{2}\left(t_{2}|\theta_{2}\right)\right),$$

where $\theta =(\theta_{1},\theta_{2})$ and ϕ is a dependence parameter.

We also assume that copula ${\tilde{C}}_{\phi }$ is given by the Ali–Mikhail–Haq copula [18]. Thus, we have

(1) $$S\left(t_{1},t_{2}|\theta ,\phi \right)={\tilde{C}}_{\phi }\left(S_{1}\left(t_{1}|\theta_{1}\right),S_{2}\left(t_{2}|\theta_{2}\right)\right)=\frac{S_{1}\left(t_{1}|\theta_{1}\right)S_{2}\left(t_{2}|\theta_{2}\right)}{1-\phi \left(1-S_{1}\left(t_{1}|\theta_{1}\right)\right)\left(1-S_{2}\left(t_{2}|\theta_{2}\right)\right)},$$

for ϕ∈[−1,1). Note that under this assumption the survival functions and the dependence structure can be visualized separately with the dependence structure represented by the copula.

Let $\left(T_{11},T_{12}\right),\cdots ,\left(T_{n1},T_{n2}\right)$ and $\left(C_{11},C_{12}\right),\cdots ,\left(C_{n1},C_{n2}\right)$ be a sample of size *n* of bivariate lifetimes and censured bivariate lifetimes, respectively. Suppose $\left(T_{i1},T_{i2}\right)$ and $\left(C_{i1},C_{i2}\right)$ are independent, for i=1,⋯,n. Consider $t_{ij}=min\left(T_{ij},C_{ij}\right)$—the *i*-th observed value and $\delta_{ij}$—a censorship indicator given by

$$\delta_{ij}=\left\{\begin{array}{cc} 1, & \mathrm{if}\;\mathrm{the}\;\mathrm{lifetime}\;\mathrm{is}\;\mathrm{uncensored},\;i.e.,T_{ij}=t_{ij};\\ 0, & \mathrm{if}\;\mathrm{the}\;\mathrm{lifetime}\;\mathrm{is}\;\mathrm{censored},\;i.e.,T_{ij}>t_{ij}, \end{array}\right.$$

for j=1,2 and i=1,⋯,n. We denote the observed values using $t=(t_{1},t_{2})$ and $\delta =(\delta_{1},\delta_{2})$, where $t_{1}=\left(t_{11},\cdots ,t_{n1}\right)$, $t_{2}=\left(t_{12},\cdots ,t_{n2}\right)$, $\delta_{1}=\left(\delta_{11},\cdots ,\delta_{n1}\right)$ and $\delta_{2}=\left(\delta_{12},\cdots ,\delta_{n2}\right)$.

The likelihood function for (θ,ϕ), given (t,δ), is (see Lawless, [19])

$$L\left(\theta ,\phi |t,\delta \right)=\prod_{i=1}^{n}f{\left(t_{i1},t_{i2}|\theta ,\phi \right)}^{\delta_{i1}\delta_{i2}}S_{\left(t_{1}\right)}^{\prime \delta_{i1}\left(1-\delta_{i2}\right)}S_{\left(t_{2}\right)}^{\prime \left(1-\delta_{i1}\right)\delta_{i2}}S{\left(t_{i1},t_{i2}|\theta ,\phi \right)}^{\left(1-\delta_{i1}\right)\left(1-\delta_{i2}\right)}$$

where $f\left(t_{i1},t_{i2}|\theta ,\phi \right)=\frac{d^{2}S\left(t_{i1},t_{i2}|\theta ,\phi \right)}{dt_{i1}dt_{i2}}$ is the joint probability density function for $\left(t_{i1},t_{i2}\right)$, $S_{\left(t_{1}\right)}^{\prime }=\left(-\frac{dS(t_{i1},t_{i2}|\theta ,\phi )}{dt_{i1}}\right)$, $S_{\left(t_{2}\right)}^{\prime }=\left(-\frac{dS(t_{i1},t_{i2}|\theta ,\phi )}{dt_{i2}}\right)$, and $S(t_{i1},t_{i2}|\theta ,\phi )$ is the copula given by (1), for i=1,⋯,n.

From Equation (1), we have

$$\begin{array}{cc} \frac{d^{2}S\left(t_{i1},t_{i2}|\theta ,\phi \right)}{dt_{i1}dt_{i2}} & =\;\frac{f_{1}\left(t_{i1}|\theta_{1}\right)f_{2}\left(t_{i2}|\theta_{2}\right)\left[\left(1+\phi \right)\left(1+\phi F_{1}\left(t_{i1}|\theta_{1}\right)F_{2}\left(t_{i2}|\theta_{2}\right)\right)-2\phi \left(F_{1}\left(t_{i1}|\theta_{1}\right)+F_{2}\left(t_{i2}|\theta_{2}\right)\right)\right]}{{\left[1-\phi F_{1}\left(t_{i1}|\theta_{1}\right)F_{2}\left(t_{i2}|\theta_{2}\right)\right]}^{3}},\\ -\frac{dS(t_{i1},t_{i2}|\theta ,\phi )}{dt_{i1}} & =\;\frac{f_{1}\left(t_{i1}|\theta_{1}\right)S_{2}\left(t_{i2}|\theta_{2}\right)\left[1-\phi F_{2}\left(t_{i2}|\theta_{2}\right)\right]}{{\left[1-\phi F_{1}\left(t_{i1}|\theta_{1}\right)F_{2}\left(t_{i2}|\theta_{2}\right)\right]}^{2}},\\ -\frac{dS(t_{i1},t_{i2}|\theta ,\phi )}{dt_{i2}} & =\;\frac{f_{2}\left(t_{i2}|\theta_{2}\right)S_{1}\left(t_{i1}|\theta_{1}\right)\left[1-\phi F_{1}\left(t_{i1}|\theta_{1}\right)\right]}{{\left[1-\phi F_{1}\left(t_{i1}|\theta_{1}\right)F_{2}\left(t_{i2}|\theta_{2}\right)\right]}^{2}}, \end{array}$$

where $F_{j}\left(t_{ij}|\theta_{j}\right)=1-S_{j}\left(t_{ij}|\theta_{j}\right)$ is the cumulative distribution function for j=1,2 and i=1,⋯,n.

### Weibull Marginal Distribution

Assume that the marginal distributions for $T_{1}$ and $T_{2}$ are given by Weibull distributions [20], i.e.,

(2) $$T_{i1}\left|\alpha_{1},\beta_{1}∼Weibull\left(\alpha_{1},\beta_{1}\right)\;\;\;\;\mathrm{and}\;\;\;\;T_{i2}\right|\alpha_{2},\beta_{2}∼Weibull\left(\alpha_{2},\beta_{2}\right),$$

with shape parameter $\alpha_{j}$ and scale parameter $\beta_{j}^{-\alpha_{j}}$ [21], each one having a probability density function

$$f\left(t_{ij}|\alpha_{j},\beta_{j}\right)=\beta_{j}\alpha_{j}t_{ij}^{\alpha_{j}-1}exp\left\{-\beta_{j}t_{i}^{\alpha_{j}}\right\}$$

for j=1,2 and i=1,⋯,n.

The survival function $S_{j}\left(t_{ij}|\theta_{j}\right)$ and hazard function $h_{j}\left(t_{ij}|\theta_{j}\right)$ are

$$S_{j}\left(t_{ij}|\theta_{j}\right)=exp\left\{-\beta_{j}t_{ij}^{\alpha_{j}}\right\}\;\;\;\;\;\mathrm{and}\;\;\;\;\;h_{j}\left(t_{ij}|\theta_{j}\right)=\beta_{j}\alpha_{j}t_{ij}^{\alpha_{j}-1}$$

respectively, where $\theta_{j}=\left(\alpha_{j},\beta_{j}\right)$ for j=1,2 and i=1,⋯,n.

Thus, the joint survival function in (1) is

$$S\left(t_{i1},t_{i2}|\theta ,\phi \right)=\frac{exp\left\{-\beta_{1}t_{i1}^{\alpha_{1}}\right\}exp\left\{-\beta_{2}t_{i2}^{\alpha_{2}}\right\}}{1-\phi \left(1-exp\left\{-\beta_{1}t_{i1}^{\alpha_{1}}\right\}\right)\left(1-exp\left\{-\beta_{2}t_{i2}^{\alpha_{2}}\right\}\right)}$$

where $\theta =(\theta_{1},\theta_{2})$. The likelihood function for (θ,ϕ) is

(3) $$L\left(\theta ,\phi |t,\delta \right)\propto \left[\prod_{j=1}^{2}\beta_{j}^{r_{j}}\alpha_{j}^{r_{j}}\exp\left\{\alpha_{j}\sum_{i=1}^{n}\delta_{ij}log\left(t_{ij}\right)-\beta_{j}\sum_{i=1}^{n}t_{ij}^{\alpha_{j}}\right\}\right]\prod_{i=1}^{n}\Psi_{i}\left(\theta ,\phi |t,\delta \right),$$

where $r_{j}=\sum_{i=1}^{n}\delta_{ij}$ is the number of uncensored observations for j=1,2, $\Psi \left(\theta ,\phi |t,\delta \right)=\prod_{k=1}^{4}\Psi_{ik}\left(\theta ,\phi |t,\delta \right)$, and

$$\begin{array}{ccc} \Psi_{i1}\left(\theta ,\phi |t,\delta \right) & = & {\left[\left(1+\phi \right)\left(1+\phi F_{1}\left(t_{i1}|\theta_{1}\right)F_{2}\left(t_{i2}|\theta_{2}\right)\right)-2\phi \left(F_{1}\left(t_{i1}|\theta_{1}\right)+F_{2}\left(t_{i2}|\theta_{2}\right)\right)\right]}^{\delta_{i1}\delta_{i2}},\\ \Psi_{i2}\left(\theta ,\phi |t,\delta \right) & = & {\left[1-\phi F_{2}\left(t_{i2}|\theta_{2}\right)\right]}^{\delta_{i1}\left(1-\delta_{i2}\right)},\\ \Psi_{i3}\left(\theta ,\phi |t,\delta \right) & = & {\left[1-\phi F_{1}\left(t_{i1}|\theta_{1}\right)\right]}^{\delta_{i2}\left(1-\delta_{i1}\right)},\\ \Psi_{i4}\left(\theta ,\phi |t,\delta \right) & = & {\left[1-\phi F_{1}\left(t_{i1}|\theta_{1}\right)F_{2}\left(t_{i2}|\theta_{2}\right)\right]}^{-(\delta_{i1}+\delta_{i2}+1)}, \end{array}$$

for i=1,⋯,n.

## 3. Bayesian Approach

In order to develop the Bayesian approach, we need to specify the prior distributions for $\alpha_{j}$, $\beta_{j}$ and ϕ, for j=1,2. We assume that priors are independent, i.e., $\pi \left(\theta ,\phi \right)=\pi \left(\theta \right)\pi \left(\phi \right)=\left[\prod_{j=1}^{2}\pi \left(\alpha_{j}\right)\pi \left(\beta_{j}\right)\right]\pi \left(\phi \right)$. Therefore, we consider the following prior distributions

$$\alpha_{j}\left|a_{j1},a_{j2}∼\Gamma \left(a_{j1},a_{j2}\right)\;\;\;\;\;\mathrm{and}\;\;\;\;\;\beta_{j}\right|b_{j1},b_{j2}∼\Gamma \left(b_{j1},b_{j2}\right),$$

where Γ(·) is the Gamma distribution and $a_{j1}$, $a_{j2}$, $b_{j1}$ and $b_{j2}$ are known hyperparameters, all of them with support on (0,+∞), for j=1,2. The parametrization of the Gamma distribution is such that the mean is $a_{j1}/a_{j2}$ and the variance is $a_{j1}/a_{j2}^{2}$, for j=1,2. The choice of values for the hyperparameters depends on the application. In the remainder of the article, we set up the hyperparameters values that give prior distributions with large variances. In particular, we set $a_{j1}=b_{j1}=0.01$, for j=1,2. For ϕ we chose the uniform prior distribution on the interval (−1,1), ϕ∼U(−1,1).

Using Bayes theorem, the joint posterior distribution for (θ,ϕ) is

π(θ,ϕ|t,δ)∝L(θ,ϕ|t,δ)π(θ)π(ϕ),

where L(θ,ϕ|t,δ) is given in Equation (3).

The conditional posterior distributions are

(4) $$\begin{array}{ccc} \pi (\alpha_{j}|t,\delta ,\theta_{-\alpha_{j}},\phi ) & \propto & \alpha_{j}^{a_{j1}+r_{j}-1}\exp\left\{\alpha_{j}\left(\sum_{i=1}^{n}\delta_{ij}log\left(t_{ij}\right)-a_{j2}\right)-\beta_{j}\sum_{i=1}^{n}t_{ij}^{\alpha_{j}}\right\}\prod_{i=1}^{n}\Psi_{i}\left(\theta ,\phi |t,\delta \right), \end{array}$$

(5) $$\begin{array}{ccc} \pi (\beta_{j}|t,\delta ,\theta_{-\beta_{j}},\phi ) & \propto & \beta_{j}^{b_{j1}+r_{j}-1}\exp\left\{-\beta_{j}\left[b_{j2}+\sum_{i=1}^{n}t_{ij}^{\alpha_{j}}\right]\right\}\prod_{i=1}^{n}\Psi_{i}\left(\theta ,\phi |t,\delta \right)\;\;\mathrm{and} \end{array}$$

(6) $$\begin{array}{ccc} \pi (\phi |t,\delta ,\theta ) & \propto & L\left(\theta ,\phi |t,\delta \right)I_{\phi }\left(-1,1\right), \end{array}$$

where $\theta_{-\nu_{j}}$, for $\nu_{j}\in \left\{\alpha_{j},\beta_{j}\right\}$, is the vector of parameters θ without the parameter $\nu_{j}$, j=1,2.

The conditional posterior distributions in Equations (4)–(6) are not familiar distributions. Thus, in order to simulate from conditional posterior distributions, we used the Metropolis–Hastings algorithm. At each iteration, the Metropolis–Hastings algorithm considers a value generated from a proposal distribution. This value is accepted according to a properly specified acceptance probability. This procedure guarantees the convergence of the Markov chain for the target density. More details on the Metropolis–Hastings algorithm can be found in [22,23,24,25] and their references.

### 3.1. MCMC for $\alpha_{j}$

Without loss of generality, we describe here how to update parameter $\alpha_{1}$ conditional on all other parameters, $\theta_{-\alpha_{1}}=\left(\beta_{1},\alpha_{2},\beta_{2}\right)$ and ϕ. The update procedure for $\alpha_{2}$ is similar.

Let $\left(\alpha_{1},\theta_{-\alpha_{1}},\phi \right)$ be the current state of the Markov chain. Consider $\alpha_{1}^{*}$ a value generated from a candidate generating density $q[\alpha_{1}^{*}|\alpha_{1}]$. The value $\alpha_{1}^{*}$ is accepted with probability $\psi \left(\alpha_{1}^{*}|\alpha_{1}\right)=min\left(1,A_{\alpha_{1}}\right)$, where

(7) $$A_{\alpha_{1}}=\frac{L\left(\alpha_{1}^{*},\theta_{-\alpha_{1}},\phi |t,\delta \right)\pi \left(\alpha_{1}^{*}\right)}{L\left(\alpha_{1},\theta_{-\alpha_{1}},\phi |t,\delta \right)\pi \left(\alpha_{1}\right)}\frac{q[\alpha_{1}|\alpha_{1}^{*}]}{q[\alpha_{1}^{*}|\alpha_{1}]},$$

and L(·|y) is the likelihood function, given in Equation (3).

The Metropolis–Hastings algorithm is implemented as follows.

- **Metropolis–Hastings Algorithm**: Let the current state of the Markov chain be $\left(\alpha_{1}^{\left(l-1\right)},\theta_{-\alpha_{1}}^{\left(l-1\right)},\phi^{\left(l-1\right)}\right)$, where *l* is the *l*-th iteration of the algorithm, $\alpha_{1}^{\left(l-1\right)}$, $\theta_{-\alpha_{1}}^{\left(l-1\right)}=\left(\beta_{1}^{\left(l-1\right)},\alpha_{2}^{\left(l-1\right)},\beta_{2}^{\left(l-1\right)}\right)$ and $\phi^{\left(l-1\right)}$ are the values of $\alpha_{1}$, $\theta_{-\alpha_{1}}$ and ϕ in (l−1)-th iteration, respectively, for l=1,⋯,L, in which, $\alpha^{\left(0\right)}$, $\theta_{-\alpha_{1}}^{\left(0\right)}$ and $\phi^{\left(0\right)}$ are the initial values. At the *l*-th iteration of the algorithm, we updated $\alpha_{1}$ as follows:
  (1) Generate $\alpha_{1}^{*}∼q\left[\alpha_{1}^{*}|\alpha_{1}\right]$;
  (2) Calculate $\psi \left(\alpha_{1}^{*}|\alpha_{1}\right)=min\left(1,A_{\alpha_{1}}\right)$, where $A_{\alpha_{1}}$ is given by (7);
  (3) Generate U∼U(0,1). If $u\leq \psi (\alpha_{1}^{*}|\alpha_{1})$ accept $\alpha_{1}^{*}$ and do $\alpha_{1}^{\left(l\right)}=\alpha_{1}^{*}$. Otherwise, reject $\alpha_{1}^{*}$ and set $\alpha_{1}^{\left(l\right)}=\alpha_{1}^{\left(l-1\right)}$.

#### 3.1.1. Two Common Choices for q[·]

To implement the Metropolis–Hastings algorithm, the candidate-generating density $q[\alpha_{1}^{*}|\alpha_{1}]$ needs to be specified. Generally, one may explore the form of the conditional posterior distribution to set the candidate-generating density. For example, if we can write $\pi (\alpha_{1}|y,\theta_{-\alpha_{1}},\phi )$ as $\pi \left(\alpha_{1}|y,\theta_{-\alpha_{1}},\phi \right)\propto \eta \left(\alpha_{1}\right)h\left(\alpha_{1}\right)$, where $h(\alpha_{1})$ is a density that can be easily generated and $\eta (\alpha_{1})$ is uniformly bounded, then we may set up $q\left(\alpha_{1}^{*}|\alpha_{1}\right)=h\left(\alpha_{1}^{*}\right)$. However, this is not the case for $\pi (\alpha_{1}|y,\theta_{-\alpha_{1}})$.

Another option is to generate $\alpha_{1}^{*}$ from a candidate generating density that does not depend on the current $\alpha_{1}$ value. That is, we may set up $q\left[\alpha_{1}^{*}|\alpha_{1}\right]=q\left[\alpha_{1}^{*}\right]$. Thus, we have a special case of the original MH algorithm, called Independent Metropolis–Hastings (IMH), where $A_{\alpha_{1}}$ is given in (7) and simplifies to

$$A_{\alpha_{1}}=\frac{L\left(\alpha_{1}^{*},\theta_{-\alpha_{1}},\phi |t,\delta \right)\pi \left(\alpha_{1}^{*}\right)}{L\left(\alpha_{1},\theta_{-\alpha },\phi |t,\delta \right)\pi \left(\alpha_{1}\right)}\frac{q[\alpha_{1}]}{q[\alpha_{1}^{*}]}.$$

In order to implement this case, one may set $q[\alpha_{1}^{*}]$ as the prior distribution, i.e., $q\left[\alpha_{1}^{*}\right]=\pi \left(\alpha_{1}^{*}\right)$. Then, $A_{\alpha_{1}}$ is given by the likelihood ratios,

(8) $$A_{\alpha_{1}}=\frac{L(\alpha_{1}^{*},\theta_{-\alpha_{1}},\phi |t,\delta )}{L(\alpha_{1},\theta_{-\alpha },\phi |t,\delta )}.$$

This algorithm is implemented as follows.

- **Independent Metropolis–Hastings Algorithm**: Let the current state of the Markov chain be $\left(\alpha_{1}^{\left(l-1\right)},\theta_{-\alpha }^{\left(l-1\right)},\phi^{\left(l\right)}\right)$. For the *l*-th iteration of the algorithm do the following:
  (1) Generate $\alpha_{1}^{*}$ from the prior distribution $\alpha_{1}^{*}∼\Gamma \left(a_{11},a_{12}\right)$;
  (2) Calculate $\psi \left(\alpha_{1}^{*}|\alpha_{1}\right)=min\left(1,A_{\alpha_{1}}\right)$, where $A_{\alpha_{1}}$ is given by (8);
  (3) Generate U∼U(0,1). If $u\leq \psi (\alpha_{1}^{*}|\alpha_{1})$ accept $\alpha_{1}^{*}$ and set $\alpha_{1}^{\left(l\right)}=\alpha_{1}^{*}$. Otherwise, reject $\alpha_{1}^{*}$ and set $\alpha_{1}^{\left(l\right)}=\alpha_{1}^{\left(l-1\right)}$.

Although the choice of the prior distribution as the candidate generating density may be mathematically attractive, it usually leads to a slow convergence of the algorithm. This happens when vague prior information is available and prior distribution has large variance. As a consequence, many of the proposed values are rejected.

An alternative is to explore the neighborhood of the current value of the Markov chain to propose a new value. This method is termed the random walk Metropolis (RWM). In the RWM, the candidate value $\alpha_{1}^{*}$ is generated from a symmetric density g(·). That is, we set up $q\left[\alpha_{1}^{*}|\alpha_{1}\right]=g(|\alpha_{1}-\alpha_{1}^{*}\left|\right)$ and the probability of generating a move from $\alpha_{1}$ to $\alpha_{1}^{*}$ depends only on the distance between them. For this case, $A_{\alpha_{1}}$ given in (7) simplifies to

(9) $$A_{\alpha_{1}}=\frac{L\left(\alpha_{1}^{*},\theta_{-\alpha_{1}},\phi |t,\delta \right)\pi \left(\alpha_{1}^{*}\right)}{L\left(\alpha_{1},\theta_{-\alpha_{1}},\phi |t,\delta \right)\pi \left(\alpha_{1}\right)}$$

since the proposal kernels from numerator and denominator cancel.

In order to implement the RWM it is necessary to simulate $\alpha_{1}^{*}$ setting $\alpha_{1}^{*}=\alpha_{1}+\varepsilon$, where ε is a random perturbation generated from a Normal distribution with mean 0 and variance $\sigma_{\alpha_{1}}^{2}$, $\varepsilon ∼N(0,\sigma_{\alpha_{1}}^{2})$, meaning that $\alpha_{1}^{*}∼N\left(\alpha_{1},\sigma_{\alpha_{1}}^{2}\right)$. This algorithm is implemented as follows.

- **Random Walk Metropolis Algorithm**: Let the current state of the Markov chain be $\left(\alpha_{1}^{\left(l-1\right)},\theta_{-\alpha_{1}}^{\left(l-1\right)},\phi^{\left(l\right)}\right)$. For the *l*-th iteration of the algorithm, l=1,⋯,L, do the following:
  (1) Generate $\varepsilon ∼N(0,\sigma_{\alpha_{1}}^{2})$ and set $\alpha_{1}^{*}=\alpha_{1}^{\left(l-1\right)}+\varepsilon$;
  (2) Calculate $\psi \left(\alpha_{1}^{*}|\alpha_{1}\right)=min\left(1,A_{\alpha_{1}}\right)$, where $A_{\alpha_{1}}$ is given by (9);
  (3) Generate U∼U(0,1). If $u\leq \psi (\alpha_{1}^{*}|\alpha_{1})$ accept $\alpha_{1}^{*}$ and set $\alpha_{1}^{\left(l\right)}=\alpha_{1}^{*}$. Otherwise, reject $\alpha_{1}^{*}$ and set $\alpha_{1}^{\left(l\right)}=\alpha_{1}^{\left(l-1\right)}$.

An issue in RWM is how to choose the value of $\sigma_{\alpha_{1}}^{2}$. It has a strong influence on the efficiency of the algorithm. If $\sigma_{\alpha_{1}}^{2}$ is too small, the random perturbations will be small in magnitude and almost all will be accepted. The consequence is that it will take a large number of iterations to explore the entire state-space. On the other hand, if $\sigma_{\alpha_{1}}^{2}$ is large there will be many rejections of the proposed values, slowing down the convergence. More details on this issue can be found in [23,26,27,28].

Typically, one may fix the value of $\sigma_{\alpha_{1}}^{2}$ by testing some values on a few pilot runs and then choosing a value whose acceptance ratio lies between 20% and 30% (see, for example, [24,25]). Thus, after a pilot run we set up $\sigma_{\alpha }^{2}=1$.

#### 3.1.2. Slice Sampling Algorithm

An alternative to the IMH and RWM sampling from some generic distribution is the slice sampling algorithm. This algorithm is a type of Gibbs sampling based on the simulation of specific uniform random variables. Here we explain the algorithm slice sampling in the context of the simulation of $\alpha_{1}$. The sampling procedure for $\alpha_{2}$ is similar. More details about SS can be found in [13].

In SS, an auxiliary variable *U* is introduced and the joint distribution $\pi (\alpha_{1},U|t,\delta ,\theta_{-\alpha_{1}},\phi )$ is given by a uniform distribution over the region $U=\{\left(\alpha_{1},u\right):0<u<\kappa \left(\alpha_{1}\right)\}$ below the curve defined by $\kappa (\alpha_{1})$. From (4), we have

(10) $$\kappa \left(\alpha_{1}\right)=\alpha_{1}^{a_{11}+r_{1}-1}\exp\left\{\alpha_{1}\left(\sum_{i=1}^{n}\delta_{i1}log\left(t_{i1}\right)-a_{12}\right)-\beta_{1}\sum_{i=1}^{n}t_{i1}^{\alpha_{1}}\right\}\prod_{i=1}^{n}\Psi_{i}\left(\theta ,\phi |t,\delta \right).$$

Marginalizing $\pi (\alpha_{1},U|t,\delta ,\theta_{-\alpha_{1}},\phi )$ over *U* yields $\pi (\alpha_{1}|t,\delta ,\theta_{-\alpha_{1}},\phi )$, so sampling from $\pi (\alpha_{1},U|t,\delta ,\theta_{-\alpha_{1}},\phi )$ and discarding *U* is equivalent to sampling from $\pi (\alpha_{1}|t,\delta ,\theta_{-\alpha_{1}},\phi )$.

As sampling from $\pi (\alpha_{1},U|t,\delta ,\theta_{-\alpha_{1}},\phi )$ is not straightforward, we implemented a Gibbs sampling algorithm where at every iteration *l*, we first generate $U^{\left(l\right)}∼U\left(0,\kappa \left(\alpha_{1}^{\left(l-1\right)}\right)\right)$ and then sample $\alpha_{1}^{\left(l\right)}∼U\left(A\right)$, where $A=\{\alpha_{1}:u^{\left(l\right)}<\kappa \left(\alpha_{1}\right)\}$. However, as the inverse of $\kappa (\alpha_{1})$ cannot be obtained analytically, we adopted the following procedure to update $\alpha_{1}$:

(i) Let λ=0.01 and $\tilde{A}$ be an empty set.
  (a) For m=1,2,…:
    Set $\alpha_{1}^{-(m)}=\alpha_{1}^{\left(l-1\right)}-m\lambda$
    If $u^{\left(l\right)}<\kappa \left(\alpha_{1}^{-(m)}\right)$ do $\tilde{A}=\tilde{A}\cup \left\{\alpha_{1}^{-(m)}\right\}$ else break
  (b) For m=1,2,…:
    Set $\alpha_{1}^{+(m)}=\alpha_{1}^{\left(l-1\right)}+m\lambda$
    If $u^{\left(l\right)}<\kappa \left(\alpha_{1}^{+(m)}\right)$ do $\tilde{A}=\tilde{A}\cup \left\{\alpha_{1}^{+(m)}\right\}$ else break
(ii) Generate $\alpha_{1}^{\left(l\right)}∼U\left(min\left(\tilde{A}\right),max\left(\tilde{A}\right)\right)$.

This algorithm is implemented as follows.

- **Slice sampling algorithm**: Let the current state of the Markov chain be $\left(\alpha_{1}^{\left(l-1\right)},\theta_{-\alpha_{1}}^{\left(l-1\right)},\phi^{\left(l-1\right)}\right)$ and $u^{\left(l-1\right)}$. For the *l*-th iteration of the algorithm, l=1,⋯,L:
  (1) Generate $U^{\left(l\right)}∼U\left(0,\kappa \left(\alpha_{1}^{\left(l-1\right)}\right)\right)$, where κ(·) is given by (10).
  (2) obtain $\tilde{A}$, conditional on $u^{\left(l\right)}$.
  (3) Generate $\alpha_{1}^{\left(l\right)}∼U\left(min\left(\tilde{A}\right),max\left(\tilde{A}\right)\right)$.

### 3.2. MCMC for $\beta_{j}$ and ϕ

Note from (5) that the conditional posterior distribution for the scale parameter $\beta_{1}$, $\pi (\beta_{1}|t,\delta ,\theta_{-\beta_{1}},\phi )$, is given by the kernel of a Gamma distribution with parameters $b_{11}+r_{11}$ and $b_{12}+\sum_{i=1}^{n}t_{i1}^{\alpha_{1}}$ multiplied by $\eta \left(\beta_{1}\right)=\prod_{i=1}^{n}\Psi_{i}\left(\theta ,\phi |t,\delta \right)$. In other words, $\pi (\beta_{1}|t,\delta ,\theta_{-\beta_{1}},\phi )$ may be written as $\pi \left(\beta_{1}|y,\theta_{-\beta_{1}}\right)\propto \eta \left(\beta_{1}\right)h\left(\beta_{1}\right)$, where $h(\beta_{1})$ is the density of the Gamma distribution $\Gamma \left(b_{11}+r_{11},b_{12}+\sum_{i=1}^{n}t_{i1}^{\alpha_{1}}\right)$ with $\eta (\beta_{1})$ being uniformly bounded. Thus, we set up the candidate generating density for $\beta_{1}$ as $q\left(\beta_{1}^{*}|\beta_{1}\right)=h\left(\beta_{1}^{*}\right)$. The acceptance probability for the generated value $\beta_{1}^{*}$ is given by $\psi \left(\beta_{1}^{*}|\beta_{1}\right)=min\left(1,A_{\beta_{1}}\right)$, where

(11) $$A_{\beta_{1}}=\frac{\eta (\beta_{1}^{*})}{\eta (\beta_{1})}.$$

This algorithm is implemented as follows.

- **Metropolis–Hastings Algorithm**: Let the current state of the Markov chain be $\left(\beta_{1}^{\left(l-1\right)},\theta_{-\beta_{1}}^{\left(l-1\right)},\phi^{\left(l-1\right)}\right)$, where $\theta_{-\beta_{1}}^{\left(l-1\right)}=\left(\alpha_{1}^{\left(l\right)},\alpha_{2}^{\left(l-1\right)},\beta_{2}^{\left(l-1\right)}\right)$. For the *l*-th iteration of the algorithm, l=1,⋯,L:
  (1) Generate $\beta_{1}^{*}∼\Gamma \left(b_{11}+r_{11},b_{12}+\sum_{i=1}^{n}t_{i1}^{\alpha_{1}^{\left(l\right)}}\right)$.
  (2) Calculate $\psi \left(\beta_{1}^{*}|\beta_{1}\right)=min\left(1,A_{\beta_{1}}\right)$, where $A_{\beta_{1}}$ is given by (11).
  (3) Generate U∼U(0,1). If $u\leq \psi (\beta_{1}^{*}|\beta_{1})$ accept $\beta_{1}^{*}$ and set $\beta_{1}^{\left(l\right)}=\beta_{1}^{*}$. Otherwise, reject $\beta_{1}^{*}$ and set $\beta_{1}^{\left(l\right)}=\beta_{1}^{\left(l-1\right)}$.

The Metropolis–Hastings algorithm for updating $\beta_{2}$ is similar. To update the dependence parameter ϕ conditional on the remaining parameters $\theta =(\alpha_{1},\beta_{1},\alpha_{2},\beta_{2})$, we used the following IMH algorithm. Let $G_{\phi }$ be a grid from −1 to 1 with increments of 0.1. Consider $\left[I_{a},I_{a+1}\right)$, an interval defined by two adjacent grid values of $G_{\phi }$ where *a* is the index of the *a*-th value of the grid for a=1,⋯,20. For example, for a=1 we have the interval [−1,−0.9); for a=11, we have the interval [0,0.1); and for a=20 we have the interval [0.9,1). Then generate the a candidate value $\phi^{*}$ as follows:

(i) If the current value of ϕ is in the interval $\left(I_{1},I_{2}\right)$, then generate $\phi^{*}$ from one of the two following Uniform distributions
  $$\phi^{*}∼\left\{\begin{array}{cc} U(I_{1},I_{2}), & \mathrm{with}\;\mathrm{probability}\;1/2,\\ U(I_{2},I_{3}), & \mathrm{with}\;\mathrm{probability}\;1/2. \end{array}\right.$$
  For this case, we generate an auxiliary variable U∼U(0,1); if u≤1/2, then we generate $\phi^{*}$ from $U(I_{1},I_{2})$, $\phi^{*}∼U\left(I_{1},I_{2}\right)$, otherwise we generate $\phi^{*}$ from $U(I_{2},I_{3})$, $\phi^{*}∼U\left(I_{2},I_{3}\right)$.
(ii) If the current value of ϕ is in $\left(I_{20},I_{21}\right)$, then generate $\phi^{*}$ from one of the two following uniform distributions
  $$\phi^{*}∼\left\{\begin{array}{cc} U(I_{19},I_{20}), & \mathrm{with}\;\mathrm{probability}\;1/2,\\ U(I_{20},I_{21}), & \mathrm{with}\;\mathrm{probability}\;1/2, \end{array}\right.$$
  Similarly to item (i), we generate an auxiliary variable U∼U(0,1); if u≤1/2, then $\phi^{*}∼U\left(I_{20},I_{21}\right)$, otherwise $\phi^{*}∼U\left(I_{19},I_{20}\right)$.
(iii) If the current value of ϕ is in the interval $\left(I_{a},I_{a+1}\right)$, for a≠1 and a≠20, then generate $\phi^{*}$ from one of three following uniform distributions
  $$\phi^{*}∼\left\{\begin{array}{cc} U(I_{a-1},I_{a}), & \mathrm{with}\;\mathrm{probability}\;1/3,\\ U(I_{a},I_{a+1}), & \mathrm{with}\;\mathrm{probability}\;1/3,\\ U(I_{a+1},I_{a+2}), & \mathrm{with}\;\mathrm{probability}\;1/3. \end{array}\right.$$
  For this case, we generate an auxiliary variable U∼U(0,1); if u≤1/3, then we generate $\phi^{*}$ from $U(I_{a-1},I_{a})$, $\phi^{*}∼U\left(I_{a},I_{a+1}\right)$; if 1/3<u≤2/3, then we generate $\phi^{*}$ from $U(I_{a},I_{a+1})$, $\phi^{*}∼U\left(I_{a},I_{a+1}\right)$; and if u>2/3, we generate $\phi^{*}$ from $U(I_{a+1},I_{a+2})$, $\phi^{*}∼U\left(I_{a+1},I_{a+2}\right)$.

The acceptance probability is given by $\psi \left[\phi^{*}|\phi \right]=min\left(1,A_{\phi }\right)$, where $A_{\phi }=\frac{L(\phi^{*},\theta |t,\delta )}{L(\phi ,\theta |t,\delta )}P_{\phi }$ for $P_{\phi }=1$ or $P_{\phi }=\frac{1/2}{1/3}$ according to items (i)–(iii) described above. This algorithm is implemented as follows.

- **IMH algorithm for ϕ**: Let the current state of the Markov chain be $\left(\theta^{\left(l\right)},\phi^{\left(l-1\right)}\right)$. For the *l*-th iteration of the algorithm, l=2,⋯,L:
  (1) Generate $\phi^{*}$ according to one of the items (i), (ii) or (iii) described above.
  (2) Calculate $\psi \left(\phi^{*}|\phi \right)=min\left(1,A_{\phi }\right)$.
  (3) Generate U∼U(0,1). If $u\leq \psi (\phi^{*}|\phi )$ accept $\phi^{*}$ and set $\phi^{\left(l\right)}=\phi^{*}$. Otherwise, reject $\phi^{*}$ and set $\phi^{\left(l\right)}=\phi^{\left(l-1\right)}$.

### 3.3. MCMC Algorithms

Using the algorithms IMH, RWM, SS and MH described above, we implemented three MCMC algorithms:

- Algorithm $A_{1}$: Parameters $\alpha_{j}$’s are updated via IMH,
- Algorithm $A_{2}$: Parameters $\alpha_{j}$’s are updated via RWM,
- Algorithm $A_{3}$: Parameters $\alpha_{j}$’s are updated via SS.

For these three algorithms, the parameters $\beta_{j}$ and ϕ are updated via MH and IMH, as described in Section 3.2, for j=1,2.

After defining the algorithms, we ran them for *L* iterations and a burn-in *B*. We also consider jumps of size *J*, i.e., only 1 drawn from every *J* was extracted from the original sequence obtaining a sub sequence of size S=[(L−B)/J] to make inferences.

The estimates for parameters are given by

(12) $${\tilde{\alpha }}_{j}=\frac{1}{S}\sum_{l=1}^{L}\alpha_{j}^{\left(K(l)\right)};\;\;\;\;\;{\tilde{\beta }}_{j}=\frac{1}{S}\sum_{l=1}^{L}\beta^{\left(K(l)\right)}\;\;\;\;\;\mathrm{and}\;\;\;\;\;\tilde{\phi }=\frac{1}{S}\sum_{l=1}^{L}\phi^{\left(K(l)\right)},$$

where $\theta^{\left(K(l)\right)}$ is the value generated for θ in the K(l)=[(B+1+lJ)]-th iteration of the algorithm, for j=1,2 and l=1,⋯,L.

## 4. Simulation Study

In this section, we present the comparison between the performances of the three algorithms applied to simulated data sets. Simulated random samples of sizes n=25,50,100 and 250 with 0%, 5%, 10%, 20% and 30% random right-censored were generated to represent small, medium and large data sets. Using these, we generated four simulated data sets with fixed parameters, as specified in Table 1.

Data set $D_{1}$ has two increasing hazard functions with a positive dependence parameter, while data set $D_{2}$ has a constant and increasing hazard function with a negative dependence parameter. Data set $A_{3}$ has parameters to produce a decreasing and a constant hazard function with weak dependence, while data set $A_{4}$ has strong dependence and two increasing hazard functions.

The simulation procedure to generate *n* observations $\left(t_{i1},t_{i2}\right)$, for i=1,⋯,n, is given by the following steps:

(i) Set up the sample size *n* and set i=1;
(ii) Generate the censoring times $C_{ij}∼U\left(0,\tau_{j}\right)$, where $\tau_{j}$ controls the percentage of censored observations, for j=1,2;
(iii) Generate uniform values $u_{ij}∼U\left(0,1\right)$, j=1,2 and calculate $w_{i}$, the solution of the nonlinear equation $u_{i2}-\frac{w_{i}\left[1-\phi \left(1-w_{i}\right)\right]}{{\left[1-\phi \left(1-u_{i1}\right)\left(1-w_{i}\right)\right]}^{2}}=0$. Here we used the *rootsolve* package and the *uniroot.all* command from *R* software to solve the nonlinear equation and obtain $w_{i}$;
(iv) Calculate $T_{i1}={\left(-\log(u_{i1})/\beta_{1}\right)}^{1/\alpha_{1}}$ and $T_{i2}={\left(-\log(w_{i})/\beta_{2}\right)}^{1/\alpha_{2}}$;
(v) Calculate the times $t_{ij}=\min\left(T_{ij},C_{ij}\right)$ and the censorship indicators $\delta_{ij}$, which are equal to 1 if $t_{ij}<T_{ij}$ and 0 otherwise, for j=1,2;
(vi) Set i=i+1. If i=n stop. Otherwise, return to step (ii).

We generated M=200 different simulated data sets according to steps (i)–(vi) described above and the parameters were estimated according to algorithms $A_{1}$, $A_{2}$ and $A_{3}$.

We used hyperparameters $a_{j1}=a_{j2}=b_{j1}=b_{j2}=0.01$ to obtain prior distributions with large variance, for j=1,2. For the *m*-th generated data set, we applied algorithms $A_{1}$, $A_{2}$ and $A_{3}$ fixing *L* = 55,000 iterations, burn-in *B* = 5000 and J=10.

Comparison of the algorithms was made using the sample Root Mean Square Error (RMSE), given by

$$\mathrm{RMSE}=\sqrt{\frac{1}{M}\sum_{m=1}^{M}\left[\sum_{j=1}^{2}{\left({\hat{\alpha }}_{j}^{\left(m\right)}-\alpha_{j}\right)}^{2}+{\left({\hat{\beta }}_{j}^{\left(m\right)}-\beta_{j}\right)}^{2}\right]+{\left({\hat{\phi }}^{\left(m\right)}-\phi \right)}^{2}}.$$

A smaller RMSE indicates better overall quality of the estimates.

Table 2 presents the RMSE value for each simulated data set by algorithm, sample size and percentage of censorship. The smaller RMSE value for each sample size and percentage of censorship is highlighted in bold. For the three algorithms, by fixing the sample size and increasing the censuring percentage (% cens.), the RMSE values increased. When the sample size increases at a fixed percentage of censures, the RMSE values decrease, consequently improving the precision of the estimators.

Based on the results presented in Table 2, for the smaller sample size n=25, the algorithm $A_{3}$ (with SS) outperformed algorithm $A_{1}$ (with IMH) and algorithm $A_{2}$ (with RWM), i.e., it gave a smaller RMSE value for all percentages of censures. This better performance also happened for data sets $D_{3}$ and $D_{4}$ for n=50. For all other simulated cases, the algorithm $A_{2}$ outperformed algorithms $A_{1}$ and $A_{3}$. An exception is the case with n=250 and 0% of censuring in data set $D_{2}$, in which algorithm $A_{1}$ had a better performance. These results suggest a possible complementarity between algorithms $A_{2}$ and $A_{3}$, where algorithm $A_{2}$ performs better for higher sample sizes and algorithm $A_{3}$ performs better for smaller sample sizes.

We verified the convergence of algorithms $A_{1}$, $A_{2}$ and $A_{3}$ using the effective sample size [14] and the integrated autocorrelation time (IAT). The effective sample size (ESS) is the number of effectively independent draws from the posterior distribution. Method with larger ESS are the most efficient. The IAT is a MCMC diagnostic that estimates the average number of autocorrelated samples required to produce one independent sample draw. Lower IAT is means more efficiency. The EES and IAT values were obtained using the *coda* and *LaplacesDemon*. Both packages are available in the *R* software.

Table A1 and Table A2 in Appendix A show the average of ESS and IAT values for each algorithm by parameter for data set $D_{1}$. Algorithm $A_{3}$ showed a better performance than algorithms $A_{1}$ and $A_{2}$, i.e., it had the highest ESS values and smallest IAT values by parameter for all simulated cases. Note that algorithm $A_{1}$ had the worst results, especially for simulated values for $\alpha_{j}$, j=1,2. Results for data sets $D_{2}$, $D_{3}$ and $D_{4}$ were similar.

**Figure A1:**
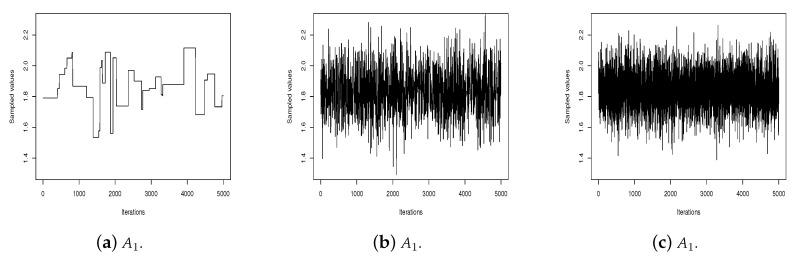

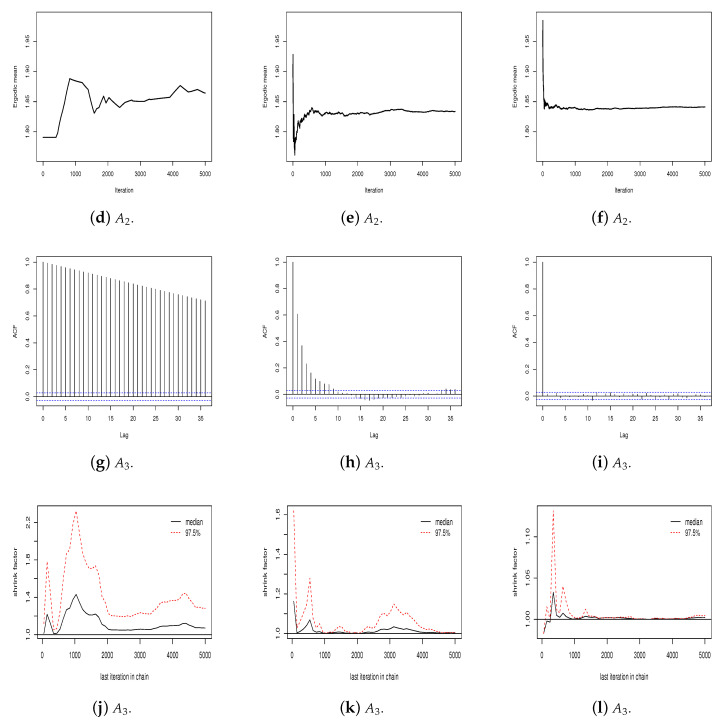
Traceplot, ergodic mean and autocorrelation for sequences produced by algorithms *A*_1_, *A*_2_ and *A*_3_ for $\alpha_{1}$.

**Figure A2:**
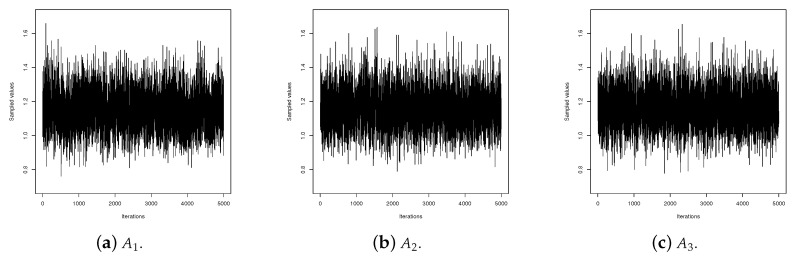

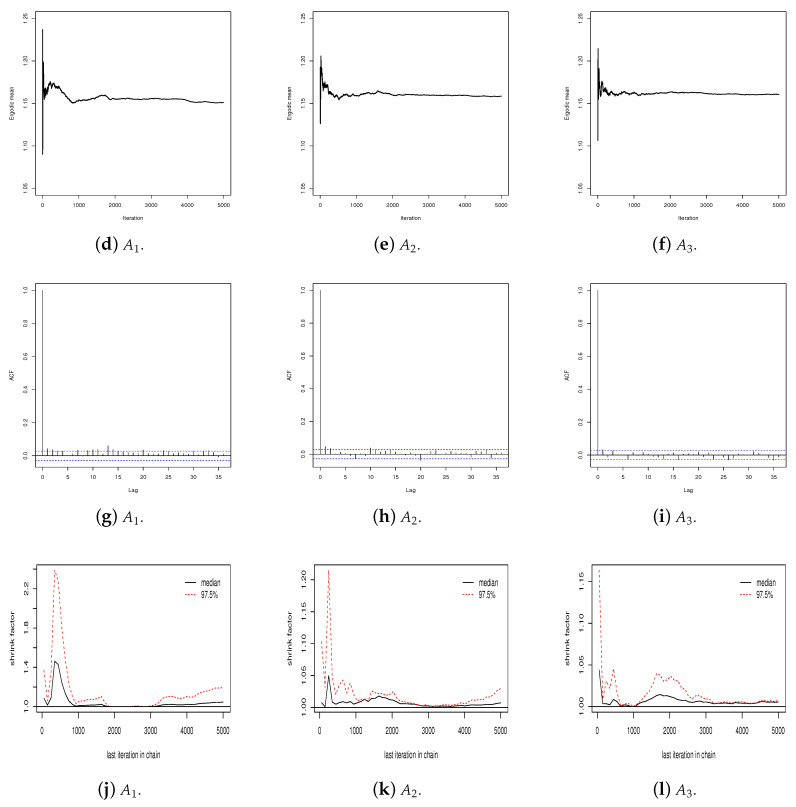
Traceplot, ergodic mean and autocorrelation for sequences produced by algorithms *A*_1_, *A*_2_ and *A*_3_ for $\beta_{1}$.

Appendix B presents an empirical convergence check for the sampled values for $\alpha_{1}$ for each algorithm. As shown in Figure A1, the generated values for $\alpha_{1}$ by algorithm $A_{1}$ did not mix well and the stability for the ergodic mean and estimated autocorrelation were not satisfactory. On the other hand, the values generated by algorithms $A_{2}$ and $A_{3}$ were well mixed and present satisfactory stability for the ergodic mean and autocorrelation. As an illustration of convergence diagnostic, Figure A1 (j–l) shows the Gelman plot for the sequence of $\alpha_{1}$ values in two chains by each algorithm. As can be seen in the figure, the number of iterations was sufficient for algorithms $A_{2}$ and $A_{3}$ to reach convergence, but not for algorithm $A_{1}$. In addition, the scale reduction factor of the Gelman–Rubin diagnostic [29] for each parameter in algorithms $A_{2}$ and $A_{3}$ were smaller than 1.1, meaning that there is no indication of non-convergence. This implies a faster convergence of algorithms $A_{2}$ and $A_{3}$ in relation to algorithm $A_{1}$. For $\beta_{1}$ sampled values, the three algorithms present satisfactory properties, i.e., good mixing, and satisfactory stability for ergodic mean and autocorrelation (see Figure A2 in Appendix B).

The results indicate that algorithm $A_{3}$ (SS for $\alpha_{j}$) is an effective alternative to algorithms $A_{1}$ (with IMH for $\alpha_{j}$) and $A_{2}$ (with RWM for $\alpha_{j}$) to simulate samples from the posterior distribution of bivariate survival models based on the Ali–Mikhail–Haq copula with marginal Weibull distributions.

## 5. Application to a Real Data Set

Next, we examine the performance of algorithms $A_{1}$, $A_{2}$ and $A_{3}$ on the diabetic retinopathy data set described in [15], which is available in the R software `survival’ package [16]. This data set consists of the follow-up times of 197 diabetic patients under 60 years of age. The main objective of the study was to evaluate the effectiveness of the photocoagulation treatment for proliferative retinopathy. The treatment was randomly assigned to one eye of each patient and the other eye was taken as a control.

Let $\left(T_{1},T_{2}\right)$ be the bivariate times, where $T_{1}$ is the time to visual loss for the treatment eye and $T_{2}$ is the time to visual loss for the control eye. The percentage of censure times for each variable is 72.59% (143 observations) for $T_{1}$ and 48.73% (96 observations) for $T_{2}$.

We used (1) to model this data with Weibull marginal distributions with parameters $\alpha_{j}$ and $\beta_{j}$ and dependence parameter ϕ.

We compared the performances of the algorithms using the RMSE in relation to the empirical distribution function,

$$\mathrm{RMSE}=\sqrt{\frac{1}{n}\sum_{i=1}^{n}\sum_{j=1}^{2}{\left({\hat{F}}_{j}\left(t_{ij}\right)-F_{j}\left(t_{ij}\right)\right)}^{2}},$$

where ${\hat{F}}_{j}\left(t_{ij}\right)$ is obtained by substituting the estimates of $\alpha_{j}$, $\beta_{j}$ and ϕ (obtained by each algorithm); and $F_{j}\left(t_{ij}\right)$ is the empirical distribution function obtained from the Kaplan–Meier estimates, for j=1,2 and i=1,⋯,n.

We ran the three algorithms using the same number of iterations, burn-in, thinning and hyperparameters values used with the simulation data. Table 3 shows the parameters estimates, the credibility intervals (95%) and RMSE values by algorithm. For this data set, the algorithm $A_{3}$ (with SS for $\alpha_{j}$) gave the smaller RMSE value.

**Figure 1:**
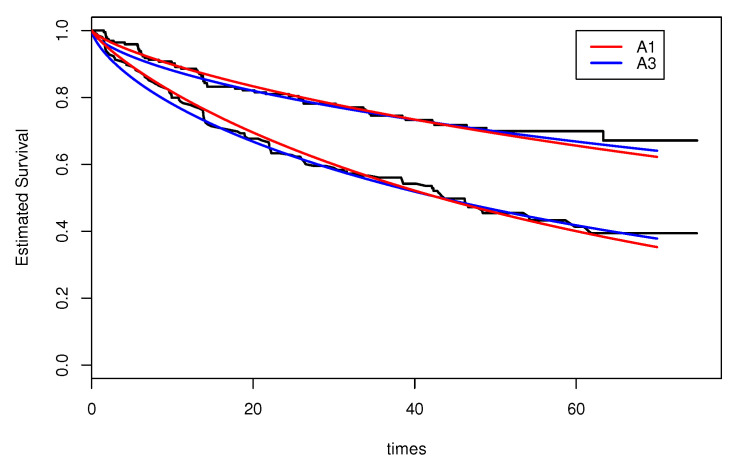
The estimated survival function for algorithms $A_{1}$ and $A_{3}$.

Figure 1 shows the estimated survival functions by algorithms $A_{1}$ (red line) and $A_{3}$ (blue line). The step functions (black lines) are the Kaplan–Meier estimates. The estimated curves by algorithms $A_{1}$ and $A_{2}$ are very close and so we show only the curve estimated by $A_{1}$, in order to provide a good visualization. The Kaplan–Meier estimates were obtained using the survival package and the survfit command in the *R* software.

Table 4 shows the ESS and IAT values for the sequences generated by algorithms $A_{1}$, $A_{2}$, and $A_{3}$. Algorithm $A_{3}$ had a better performance than algorithms $A_{1}$ and $A_{2}$, i.e., the highest ESS value and the lowest IAT value per parameter.

**Figure 2:**
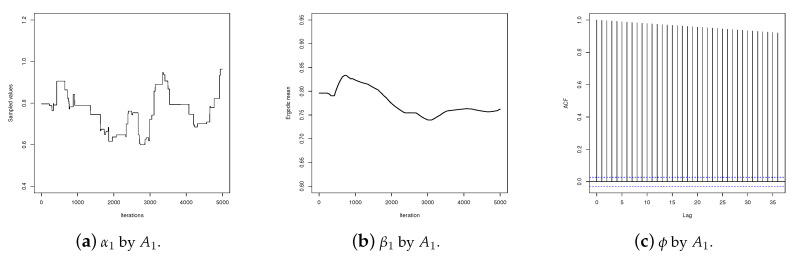

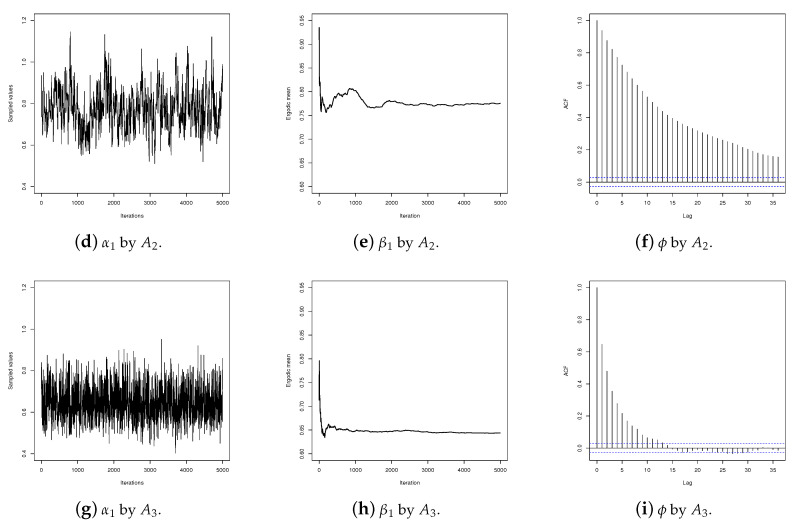
Traceplot, ergodic mean and autocorrelation for sequences produced by algorithms *A*_1_, *A*_2_ and *A*_3_ for $\alpha_{1}$.

We also compared the performances of the algorithms in relation to the sequences generated for each parameter. Figure 2 shows the traceplots, the ergodic means, and the autocorrelations for sequences of $\alpha_{1}$ values simulated by algorithms $A_{1}$, $A_{2}$ and $A_{3}$.

**Figure 3:**
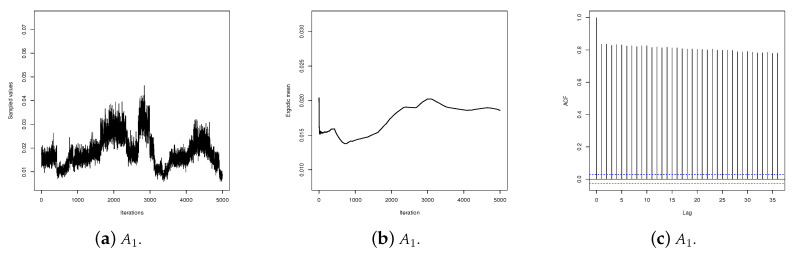

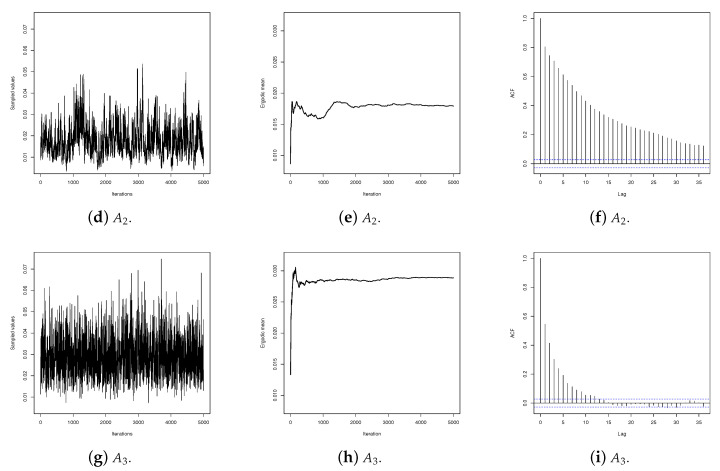
Traceplot, ergodic mean and autocorrelation for sequences produced by algorithms *A*_1_, *A*_2_ and *A*_3_ for $\beta_{1}$.

It can be observed in these graphs that the $\alpha_{1}$ values generated by the IMH (algorithm $A_{1}$) has poor mixing, does not show satisfactory stability for the ergodic mean, and the autocorrelation is high for long lags. On the other hand, the values generated by the RWM (algorithm $A_{2}$) and SS (algorithm $A_{3}$) are better mixed and present satisfactory stability for the ergodic mean. However, the sequence produced by the SS presents the steepest decreasing autocorrelation. Figure 3 shows the same graphs for parameter $\beta_{1}$. As can be seen, for $\beta_{1}$ the performances of the three algorithms are satisfactory. These results, together with those presented by the RMSE, show that for the data set analyzed here SS provides a better performance than IMH or RWM.

**Figure 4:**
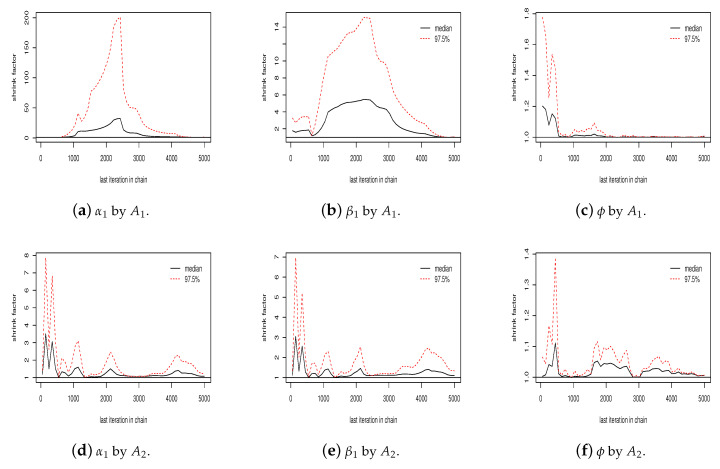

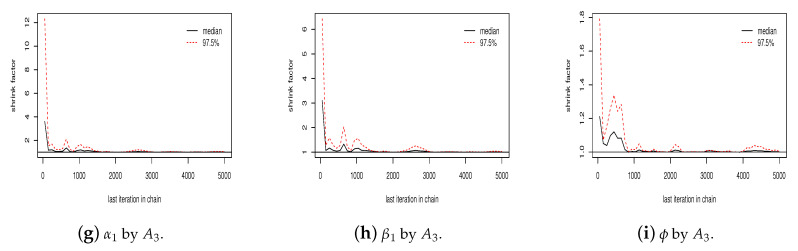
Gelman plot for two sequences produced by algorithms *A*_1_, *A*_2_ and *A*_3_ for $\alpha_{1}$, $\beta_{1}$ and ϕ.

Figure 4 shows the Gelman plot for the simulated values for $\alpha_{1}$, $\beta_{1}$ and ϕ in two chains by each algorithm. As can be seen, the number of iterations was sufficient for algorithms $A_{2}$ and $A_{3}$ to reach the convergence, but not sufficient for algorithm $A_{1}$ (Figure 4a,b). The scale reduction factor for each parameter in algorithms $A_{2}$ and $A_{3}$ are all less than 1.1, while for algorithm $A_{1}$ only ϕ presents a scale reduction factor less than 1.1.

## 6. Final Remarks

We investigated the performances of three Bayesian computational methods to estimate parameters of a bivariate survival model based on the Ali–Mikhail–Haq copula with marginal Weibull distributions. The performances of the MCMC algorithms were compared using the RMSE criterion. The RMSE values were calculated for different sample sizes and different percentages of censures.

The results obtained from the simulated data sets showed that the RWM and SS algorithms outperformed the IMH algorithm, and that the SS algorithm performed better for lower sample sizes. The results show evidence that MCMC sequences obtained with SS with the same number of iterations *L*, *burn in* *B* and thinning value, have better properties (i.e., higher ESS and lower IAT values) than for IMH and RWM, which are standard methods to sample from the joint posterior distribution.

We also illustrate the application of the algorithms using a real data set, available in the literature. The algorithm $A_{3}$ (with SS generating the $\alpha_{j}$’s) presented a better performance when applied to this data set. The criteria used to reach this conclusion were the stability for the ergodic mean, the autocorrelation, the minimum RMSE value, the maximum ESS value, and the minimum IAT value. In addition, the algorithm using SS presented a satisfactory performance in relation to scale factor reduction, and the Gelman plot of the Gelman–Rubin convergence diagnostic.

Our results show that algorithm $A_{3}$, which is composed by a mixing of SS for generating $\alpha_{j}$, MH for $\beta_{j}$ and IMH for ϕ, is an effective algorithm to simulate values from the joint posterior distribution of an AMH copula with Weibull marginal distributions. Moreover, two advantages of SS are that it is easy to implement and it does not need to specify a candidate generating density. A disadvantage in our specific case is that it took longer to perform the simulation when compared with IMH and RWM. The reason for this longer time is that we needed an iterative method to obtain the inverse of the function $\kappa (\alpha_{j})$. This was because an analytical solution is not available. All calculations were implemented using the software *R* and can be obtained from the authors.

An extension of the results obtained here for other Arquimedian copulas as well other marginal distributions and a possible generalization would be a fruitful area for future work. 

## Figures and Tables

**Table 1:** Parameter values for simulated data sets.

Data Set	Parameters				
	$\alpha_{1}$	$\beta_{1}$	$\alpha_{2}$	$\beta_{2}$	ϕ
$D_{1}$	2.00	1.00	3.00	1.00	0.50
$D_{2}$	1.00	2.00	2.00	0.50	−0.75
$D_{3}$	0.75	1.50	1.00	2.00	0.05
$D_{4}$	1.80	2.40	2.20	1.20	0.95

**Table 2:** Root mean square error (RMSE) by algorithm for data sets $D_{1}$, $D_{2}$, $D_{3}$ and $D_{4}$.

Sample Size	% of Censures	Data Set $D_{1}$			Data Set $D_{2}$			Data Set $D_{3}$			Data Set $D_{4}$		
		Algorithm			Algorithm			Algorithm			Algorithm		
		$A_{1}$	$A_{2}$	$A_{3}$	$A_{1}$	$A_{2}$	$A_{3}$	$A_{1}$	$A_{2}$	$A_{3}$	$A_{1}$	$A_{2}$	$A_{3}$
n=25	0%	0.3678	0.3717	**0.3581**	0.3774	0.3781	**0.3458**	0.3375	0.3370	**0.3368**	1.1085	1.0888	**1.0883**
	5%	0.4078	0.3869	**0.3597**	0.3861	0.3901	**0.3736**	0.3586	0.3573	**0.3523**	1.1325	1.1305	**1.1278**
	10%	0.4189	0.4012	**0.3670**	0.4144	0.4259	**0.4135**	0.3687	0.3675	**0.3611**	1.1428	1.1396	**1.1323**
	20%	0.4245	0.4153	**0.3772**	0.4472	0.4648	**0.4381**	0.3772	0.3729	**0.3727**	1.1726	1.1714	**1.1711**
	30%	0.4362	0.4543	**0.3989**	0.5335	0.5614	**0.5303**	0.3994	0.3990	**0.3944**	1.2078	1.1946	**1.1925**
n=50	0%	0.2595	**0.2507**	0.2678	0.2633	**0.2552**	0.2573	0.2162	0.2112	**0.2048**	1.0397	1.0318	**1.0312**
	5%	0.2663	**0.2652**	0.2699	0.2641	**0.2601**	0.2719	0.2239	0.2283	**0.2233**	1.0470	1.0442	**1.0403**
	10%	0.2831	**0.2806**	0.2814	0.2959	**0.2683**	0.2844	0.2390	0.2457	**0.2269**	1.0483	1.0453	**1.0433**
	20%	0.2846	**0.2820**	0.2863	0.2966	**0.2820**	0.3026	0.2719	0.2546	**0.2366**	1.0517	1.0528	**1.0513**
	30%	0.2983	**0.2885**	0.3104	0.3245	**0.3170**	0.3182	0.2828	0.2776	**0.2736**	1.0915	1.0666	**1.0550**
n=100	0%	0.1822	**0.1819**	0.1833	0.1917	**0.1816**	0.1878	0.1664	**0.1657**	0.1702	1.0153	**1.0041**	1.0124
	5%	0.1953	**0.1851**	0.1859	0.1925	**0.1857**	0.1914	0.1769	**0.1755**	0.1782	1.0228	**1.0063**	1.0152
	10%	0.1982	**0.1924**	0.1927	0.2026	**0.2019**	0.2023	0.1788	**0.1760**	0.1791	1.0239	**1.0088**	1.0157
	20%	0.1996	**0.1964**	0.2074	0.2029	**0.2028**	0.2047	0.1934	**0.1832**	0.1879	1.0282	**1.0092**	1.0177
	30%	0.2131	**0.2122**	0.2144	0.2463	**0.2112**	0.2211	0.2094	**0.1967**	0.2143	1.0291	**1.0128**	1.0265
n=250	0%	0.1138	**0.1123**	0.1130	**0.1075**	0.1079	0.1115	0.1156	**0.1140**	0.1162	0.9934	**0.9923**	0.9936
	5%	0.1141	**0.1136**	0.1149	0.1206	**0.1141**	0.1129	0.1179	**0.1146**	0.1183	0.9970	**0.9963**	0.9968
	10%	0.1165	**0.1164**	0.1167	0.1244	**0.1199**	0.1237	0.1186	**0.1159**	0.1197	0.9985	**0.9977**	0.9972
	20%	0.1224	**0.1216**	0.1229	0.1258	**0.1252**	0.1287	0.1303	**0.1260**	0.1273	0.9991	**0.9984**	0.9991
	30%	0.1374	**0.1333**	0.1344	0.1677	**0.1398**	0.1458	0.1391	**0.1328**	0.1329	0.9999	**0.9993**	0.9997

**Table 3:** Parameters estimates and RMSE by algorithm.

Algorithm	Parameter					RMSE
	$\alpha_{1}$	$\beta_{1}$	$\alpha_{2}$	$\beta_{2}$	ϕ	Value
$A_{1}$	0.7624	0.0186	0.8399	0.0294	0.7159	0.4227
	(0.5999,0.9361)	(0.0087, 0.0338)	(0.7607, 0.9353)	(0.0195, 0.0414)	(0.3765, 0.9637)	
$A_{2}$	0.7757	0.0179	0.8308	0.0310	0.7148	0.4619
	(0.5929, 0.9853)	(0.0071, 0.0343)	(0.6897, 0.9679)	(0.0172, 0.0515)	(0.3560, 0.9600)	
$A_{3}$	0.6438	0.0289	0.7015	0.0494	0.7266	0.3562
	(0.5103, 0.7967)	(0.0142, 0.0482)	(0.5910, 0.8273)	(0.0293, 0.0746)	(0.3675, 0.9715)	

**Table 4:** Integrated autocorrelation time (IAT) and effective sample size (ESS) values for algorithms $A_{1}$, $A_{2}$ and $A_{3}$.

Parameter	ESS			IAT		
	$A_{1}$	$A_{2}$	$A_{3}$	$A_{1}$	$A_{2}$	$A_{3}$
$\alpha_{1}$	5.4650	159.8655	791.0559	435.0485	34.2212	6.4039
$\beta_{1}$	6.5887	205.4812	880.9221	81.9980	26.8373	5.6359
$\alpha_{2}$	8.1633	134.7412	227.6705	327.9376	35.6760	24.6754
$\beta_{2}$	16.1893	133.8282	230.9487	36.7590	30.5560	21.1668
ϕ	2443.3791	2400.0097	2461.1781	2.3426	2.3348	2.2813

## Appendix A. ESS and IAT Values for Simulated Data Sets

In this section, we present the average of ESS and IAT values for each algorithm by parameter for data set $D_{1}$. As discussed in Section 4, Algorithm $A_{3}$ presented a better performance than algorithms $A_{1}$ and $A_{2}$. The results for data sets $D_{2}$, $D_{3}$ and $D_{4}$ are similar.

**Table A1:** ESS by algorithm for data sets $D_{1}$.

Sample Size	% of Censures	Algorithm $A_{1}$					Algorithm $A_{2}$					Algorithm $A_{3}$				
		$\alpha_{1}$	$\beta_{1}$	$\alpha_{2}$	$\beta_{2}$	ϕ	$\alpha_{1}$	$\beta_{1}$	$\alpha_{2}$	$\beta_{2}$	ϕ	$\alpha_{1}$	$\beta_{1}$	$\alpha_{2}$	$\beta_{2}$	ϕ
n=25	0%	25.4	1149.9	26.0	1168.4	105.9	1741.7	3493.7	1816.3	3511.8	111.2	4547.7	4110.0	4540.0	4136.9	112.2
	5%	26.4	1360.4	27.4	1311.1	100.6	1758.1	3530.2	1823.3	3563.4	106.8	4569.7	4118.5	4622.4	4125.7	112.0
	10%	27.9	1570.5	28.2	1422.5	97.6	1783.3	3543.0	1827.7	3598.9	99.9	4604.9	4220.7	4672.7	4191.9	105.2
	20%	31.8	2178.7	30.1	1988.6	95.6	1869.0	3943.1	1822.2	3738.9	93.9	4681.8	4275.1	4726.5	4182.3	97.9
	30%	32.9	2293.8	32.7	2146.3	88.5	1931.0	4018.4	1772.0	3885.7	88.1	4782.5	4350.3	4744.4	4329.9	89.6
n=50	0%	19.4	860.7	19.5	1049.2	173.0	1415.2	3259.1	1774.8	3450.9	172.7	4607.70	4132.9	4610.4	4129.5	176.9
	5%	19.6	1061.1	18.7	968.2	167.2	1475.8	3456.2	1796.2	3517.1	167.3	4680.2	4226.3	4698.9	4187.6	169.3
	10%	21.1	1331.7	20.6	1168.2	163.2	1565.6	3662.3	1861.4	3700.1	155.8	4706.1	4237.6	4698.8	4148.0	171.4
	20%	22.5	2134.5	23.1	2005.2	141.6	1668.8	3926.3	1922.5	3804.2	140.0	4825.1	4374.9	4792.8	4299.3	143.6
	30%	24.3	2604.9	24.5	2241.4	127.0	1770.5	4188.2	1989.0	4047.5	132.2	4817.7	4504.1	4819.8	4364.1	133.8
n=100	0%	14.3	817.5	14.8	826.7	316.7	1107.5	3258.6	1518.9	3429.5	323.9	4609.3	4244.3	4668.7	4169.3	325.2
	5%	14.5	899.7	14.5	807.8	304.1	1136.7	3393.6	1549.6	3522.7	290.0	4639.9	4238.7	4689.2	4222.8	311.4
	10%	15.6	1157.9	15.0	938.3	276.9	1199.2	3617.4	1598.7	3698.5	272.9	4729.9	4311.9	4800.5	4295.0	277.3
	20%	16.3	1846.4	16.4	1540.7	260.7	1297.1	3886.4	1706.2	3834.2	265.2	4833.4	4465.1	4827.2	4399.4	271.4
	30%	17.6	3127.3	17.7	2337.1	224.4	1414.1	4292.0	1831.9	4128.8	211.1	4857.6	4475.2	4862.9	4410.8	226.3
n=250	0%	10.3	655.3	10.0	662.7	672.9	712.3	2856.1	1055.4	3236.4	687.8	4588.1	4210.6	4655.5	4275.5	698.8
	5%	10.7	800.5	10.5	816.3	672.3	742.5	3106.1	1083.3	3343.3	640.0	4664.5	4333.8	4734.3	4277.8	693.9
	10%	10.7	1024.2	10.8	951.7	602.3	786.7	3369.7	1128.4	3519.9	607.5	4728.8	4362.8	4757.3	4338.3	620.0
	20%	10.7	1735.2	11.8	1494.5	549.7	863.0	3890.0	1226.9	3845.6	539.6	4741.7	4440.4	4805.1	4451.7	550.0
	30%	12.2	3259.7	12.1	2271.8	466.2	936.6	4279.2	1308.9	4147.7	477.2	4872.7	4625.0	4858.4	4552.6	481.6

**Table A2:** IAT by algorithm for data sets $D_{1}$.

Sample Size	% of Censures	Data Set $A_{1}$					Data Set $A_{2}$					Data Set $A_{3}$				
		$\alpha_{1}$	$\beta_{1}$	$\alpha_{2}$	$\beta_{2}$	ϕ	$\alpha_{1}$	$\beta_{1}$	$\alpha_{2}$	$\beta_{2}$	ϕ	$\alpha_{1}$	$\beta_{1}$	$\alpha_{2}$	$\beta_{2}$	ϕ
n=25	0%	162.7	2.4	162.4	2.3	50.6	3.0	1.5	2.9	1.5	50.2	1.1	1.3	1.1	1.2	50.0
	5%	162.3	2.2	154.0	2.3	52.5	2.9	1.5	2.8	1.5	50.2	1.1	1.2	1.1	1.2	50.0
	10%	152.7	2.0	150.9	2.3	54.1	2.9	1.5	2.8	1.5	54.8	1.1	1.2	1.1	1.2	51.3
	20%	136.8	1.7	136.6	1.9	55.4	2.7	1.3	2.8	1.4	55.8	1.1	1.2	1.1	1.2	54.5
	30%	132.2	1.7	130.4	1.7	59.9	2.6	1.3	3.0	1.4	59.8	1.1	1.2	1.1	1.2	57.6
n=50	0%	208.9	2.3	213.5	2.2	33.2	3.7	1.6	2.9	1.5	32.8	1.1	1.2	1.1	1.2	32.5
	5%	208.7	2.0	233.6	2.2	34.8	3.5	1.5	2.9	1.5	34.5	1.1	1.2	1.1	1.2	34.2
	10%	198.6	1.9	206.5	2.2	35.6	3.3	1.4	2.7	1.4	36.0	1.1	1.2	1.1	1.2	35.2
	20%	183.6	1.6	179.4	1.6	39.5	3.1	1.3	2.7	1.4	39.2	1.1	1.2	1.1	1.2	39.0
	30%	170.5	1.5	170.0	1.6	43.2	2.9	1.2	2.5	1.3	41.9	1.1	1.1	1.1	1.2	40.3
n=100	0%	288.1	2.1	278.2	2.2	17.9	4.6	1.6	3.4	1.5	18.1	1.1	1.2	1.1	1.2	17.2
	5%	284.7	2.2	287.2	2.2	19.7	4.5	1.5	3.3	1.5	20.3	1.1	1.2	1.1	1.2	18.9
	10%	266.8	1.9	271.9	1.9	21.3	4.2	1.4	3.2	1.4	20.5	1.1	1.2	1.1	1.2	20.3
	20%	250.0	1.6	252.8	1.7	22.8	3.9	1.4	3.0	1.4	22.4	1.1	1.1	1.1	1.2	22.3
	30%	233.4	1.3	227.1	1.5	26.5	3.6	1.2	2.8	1.2	27.0	1.1	1.1	1.1	1.2	26.2
n=250	0%	417.9	2.0	418.8	2.0	7.9	7.1	1.8	4.8	1.6	7.9	1.1	1.2	1.1	1.2	7.6
	5%	400.6	1.9	399.7	2.0	8.2	6.8	1.7	4.7	1.6	8.4	1.1	1.2	1.1	1.2	8.1
	10%	391.7	1.8	366.7	1.8	9.1	6.5	1.5	4.5	1.5	9.0	1.1	1.2	1.1	1.2	8.8
	20%	374.6	1.5	355.9	1.6	10.2	5.9	1.3	4.1	1.4	10.3	1.1	1.2	1.1	1.2	10.1
	30%	358.9	1.3	339.2	1.4	11.8	5.5.	1.5	3.9	2.1	11.7	1.1	1.1	1.1	1.1	11.1

## Appendix B. Empirical Illustration of the Convergence

We present here an empirical illustration of the convergence of the simulated sequences for parameters $\alpha_{1}$ and $\beta_{1}$. We randomly selected a data set from one of the M=200 generated data sets $D_{1}$ with n=100 and %cens=5 and present the traceplot, graphs showing of the ergodic mean and autocorrelation of the sampled values by algorithm and the Gelman plot.

Figure A1 shows the performance of the algorithms for sampled $\alpha_{1}$ values. It can be observed that the IMH (algorithm $A_{1}$) does not mix well, it does not have stability for the ergodic mean, and the estimated autocorrelation does not decrease as fast as the other algorithms. The sequences of $\alpha_{1}$’s generated by RWM and SS are well mixed and present satisfactory stability for the ergodic mean, and the autocorrelation decreases faster, with a clear advantage for algorithm $A_{3}$. The Gelman plot indicates that the number of iterations used was sufficient for algorithms $A_{2}$ and $A_{3}$ to reach the convergence.

Figure A2 presents the performances of each algorithm for the sequence generated for $\beta_{1}$. As can be observed, the three algorithms present satisfactory properties. The satisfactory performance of the three algorithms is mainly due to the fact that $\beta_{1}$ has a natural candidate generating density with parameters depending on the observed data and values of hyperparameters.
